# The price of longevity

**DOI:** 10.18632/aging.104215

**Published:** 2020-11-28

**Authors:** Nico P. Dantuma, Thorsten Hoppe, Laura K. Herzog

**Affiliations:** 1 Department of Cell and Molecular Biology, Karolinska Institutet, Stockholm, S-17177, Sweden; 2 Institute for Genetics and Cologne Excellence Cluster on Cellular Stress Responses in Aging-Associated Diseases (CECAD), Cologne, Germany; 3 Center for Molecular Medicine Cologne (CMMC), University of Cologne, Cologne, Germany

**Keywords:** autophagy, ubiquitin, ataxin-3, proteostasis, longevity

Why some people live a healthy long life whereas others are less fortunate is one of those unsolved mysteries fascinating scientists and non-scientists alike. The desire to stay young in our modern Western culture is reflected in the numerous healthy diets and lifestyles that have been advocated to increase longevity. The molecular pursuit of this Holy Grail has resulted in the discovery of several cellular hallmarks of aging, including the decline in protein homeostasis (i.e. proteostasis) that can cause increased aggregation of dysfunctional proteins, which is detrimental to cells and affects organismal metabolism [1]. Proteostasis is supported by autophagy, a conserved machinery that helps to eliminate dysfunctional proteins and cellular organelles via lysosomal degradation. The rejuvenating power of cleaning up garbage and replacing it with recycled and newly synthesized cellular components speaks to the imagination and is an attractive explanation for the positive correlation between autophagy and lifespan. This connection is highly conserved and well reflected by research utilizing model organisms that confirms the positive impact of increased autophagy on aging in yeast, worms, flies, zebrafish, and mice. The life expanding potential of dietary restriction can also be linked to autophagy as this is known to stimulate nutrient recycling. Conversely, genetic defects in autophagy are frequently associated with decreased lifespan and higher risk for chronic age-related diseases including neurodegenerative disorders.

Early studies suggesting that longevity is regulated by autophagy took advantage of *Caenorhabditis elegans*, a genetically tractable model organism with a short lifespan. More recent work in *C. elegans* identified that another link between longevity and proteostasis is mediated by the ubiquitin-proteasome system (UPS), a proteolytic pathway that degrades damaged proteins via the proteasome [2]. Worms lacking the ubiquitin-selective unfoldase CDC-48.1 and the ubiquitin deconjugase ATX-3 exhibit a significantly increased lifespan. Besides protein degradation by the UPS, both proteins have also been shown to be important for autophagy [3,4]. This may not be surprising as the UPS and autophagy are not only the two main intracellular proteolytic systems but also highly interconnected and functionally complementary. This remarkable molecular interconnection raised questions about the functional status of the UPS and autophagy in these long-lived worms lacking CDC-48.1 and ATX-3. Our recent work resulted in the intriguing observation that the increased lifespan comes at a cost: while the *cdc-48.1; atx-3* double mutant worms live longer they have to carry the burden of an increased sensitivity towards starvation [5]. This phenomenon suggests a defect in autophagy as this process is critical in recycling nutrients in the absence of food. Thus, contrary to the increase in autophagy observed in other long-lived mutants, autophagy appeared to be impaired in these worms. Further analysis revealed that the increased starvation sensitivity of these mutants mainly depends on the role of the ubiquitin deconjugase ATX-3 in autophagy regulation while lack of CDC-48.1 does not compromise starvation survival.

In line with these results, we indeed found a decrease in autophagy flux upon depletion of the human ATX-3 orthologue Ataxin-3 in cell culture models [5]. While Ataxin-3 deficient cells are still able to induce autophagy, the induction process appeared to be exaggerated with an increased number of autophagosomes, which is accompanied by less efficient turnover of proteins by autophagy. Moreover, Ataxin-3 interacts with the autophagosomal membrane proteins LC3C and GABARAP and transiently localized to early autophagosomes. Our observations raised the idea that Ataxin-3 mediated deubiquitylation might locally suppress the formation of autophagosomes thereby enhancing the efficacy of the autophagy flux. Regardless the mechanism underlying the conserved role of ATX-3/Ataxin-3 in autophagy function, our work unequivocally shows that longevity can be accompanied by a decrease rather than an increase in autophagy. In this context it is noteworthy that a repeat expansion in the Ataxin-3 gene is responsible for the age-related, neurodegenerative disorder Machado Joseph disease (MJD), also known as spinocerebellar ataxia type 3 (SCA-3) [6]. Another repeat expansion protein, namely huntingtin, the protein causative for Huntington’s disease, has been found to play a pivotal role in substrate recruitment of autophagosomes [7]. Both diseases are characterized by an accumulation of misfolded proteins in ubiquitin-labelled intraneuronal inclusion bodies, structures that are frequently observed in autophagy-deficient mice [8]. The newly discovered role of Ataxin-3 raises the question to what extent the toxic accumulation of misfolded proteins in SCA-3 is a consequence of inefficient intracellular clearance of aggregates due to defective autophagy. The identification of Ataxin-3 substrates and its role in autophagy regulation await further experimental clarification. For now, we can conclude that, as the saying goes: Everything comes at a price …. even longevity.
